# The Orphan Working School

**Published:** 1892-05-21

**Authors:** 


### II.—THE ORPHAN WORKING SCHOOL.

The 134th anniversary festival of the Orphan Working School was held at the Whitehall Rooms on the 13th inst. This School admits orphan and necessitous children from any part of the United Kingdom, providing they are under eleven years of age, and the children are clothed, maintained, educated, and fitted for making their way in life. Each boy or girl on leaving the school is given a sum of money to provide an outfit, and during the next seven years they are eligible for the prizes given for good conduct in their respective situations. That great interest is taken in the work of the institution was shown by the large number of ladies and gentlemen who attended the dinner on the 13th inst., and by the good result of the appeal now being made for funds. Altogether £8,000 is required to get the institution out of debt. With the able Secretary, Mr. A. P. Coote, we hope that the collecting cards which the ladies were given during the evening will bring in the £4,000 still required to place a charity, which has for much over a century been doing a great work among the fatherless and widows, "out of debt," and, therefore, if our copy-books are right, "out of danger" of having, from lack of funds, to lessen in any way its usefulness. A very pleasant evening was the result of Mr. Coote's labours in regard to this year's festival dinner, and a word of praise is due to the Meister Glee Singers, who did much to render the after-dinner part of the proceedings enjoyable.

The Chairman (Mr. Francis A. Bevan, D.L.) in proposing the toast of the evening—"Prosperity to the Orphan Working School"—said that in no other countries in the world, except England and the United States, were large institutions maintained simply out of the kindness of heart and the charity of men and women. No institution appealed to one's sympathies so much as an orphan institution. The occupants of other institutions might possibly be able to help themselves in some measure, but little children deprived of their only guardians and supporters, but for the kindness and sympathy of Christian men and women, must be left to perish or to grow up in ignorance. Of all such institutions in this country the Orphan Working School was the oldest. It was born, so to speak, on May 10th, 1758, so that that evening they were celebrating its 134th birthday. At the present time this school ought to be an especial object of sympathy to all, for during the past year it had gone through exceptional trials. Two epidemics of sickness had broken out, first influenza, and then scarlet fever, no less than 42 of the children being laid up with the latter. All these children were sent to the Fever Hospital, Liverpool Road, but the management were not content to let things rest there, and determined to go to the root of the matter. Acting on the advice of their medical officers, they went thoroughly into the subject of the drainage, and had it carefully overhauled, with the result that it had cost them about £5,000. All this work had necessitated the boarding out of the children, which also had added considerably to the special expenditure of the year. A correspondent had written complaining that they spent £18,000 a year, and only maintained 600 children. How many children this correspondent expected them to maintain he did not say, but the letter had caused him (the Chairman) to compare their expenditure with that of other similar institutions, and he found that all of them came out at about the same amount. Therefore, considering that the committees of all of these institutions were composed of business men, accustomed to look at things in a business light, and also considering that they had to properly educate, maintain, clothe, and fit the children in their charge for the position they were to occupy in life, he had come to the conclusion that there was no extravagance at all, and that the money spent was only what was right and proper. The result of their work was most gratifying, for 98 per cent. of the children who were trained in the Orphan Working School were said to do well in after life. Employers in the City liked to get boys from their school, for they knew they had been not merely ornamented with certain technicalities of knowledge, but that they had been also grounded in sound principles that would remain with them all their lives.

The Treasurer (Mr. B. Woodd Smith) proposed "The Health of the Chairman," and said that this was the very time when the institution wanted the help of all its friends. (Applause.)

The Chairman having briefly responded,

Mr. Burdett proposed "The Health of the Treasurer, Committees, and Stewards." Unless these officers, he said, had the utmost public spirit and gave the utmost care and knowledge to the management of the institution in their charge they were not a blessing but something much worse. During the past eighteen months he had had his attention drawn somewhat closely to what were known as benevolent institutions as opposed to medical institutions, and he thought that might be a fitting occasion to try and help them by dealing with certain points which he regarded as of the utmost importance to the management of the Orphan Working School. There was a shyness about benevolent institutions which was not to be found in the management of the medical institutions of this country. Both classes of institutions depended for the greater portion of their receipts upon voluntary contributions, and, therefore, if the managers of benevolent institutions were wise they would turn the utmost public light on to their work. Unfortunately, for some unascertainable reason which he was utterly unable to account for, he did find that compared with hospitals the benevolent institutions were asleep as to the importance of the public eye, rather shunning than seeking it. One result of their dinner would be, he hoped, that the Orphan Working School would seek publicity, and if it did so he felt sure that great and good as had been its work in the past, its work in the future would be still greater and better. There was, however, one kind of publicity which was painful to him, and that was the parading of the children in the school at the festival dinner. If anyone wanted to see them they could go to the school, and he did not think it was right to bring young children out at that late hour, nor was that sort of publicity worthy of the boasted intelligence of the nineteenth century. He was very strongly of opinion that as the utmost good had resulted from an interchange of opinion and knowledge in regard to the administration and management of medical charities, so a similar amount of good would accrue from the same system in regard to the orphanages of this country. As they had now a very able and energetic Secretary who had been trained under the system of the medical institutions, they had already at hand a means to this end, which it would be well to make use of. It was of the utmost importance also to the work done at our orphanages that it should be possible to retain the children in the institutions until they were able to support themselves. One of the faults and drawbacks, one of the causes of the failure of the English system of district schools at the present time, was that the law did not give the guardians power to keep the children long enough at school. And he did think, and he was glad to hear from one of them that that view prevailed amongst the Committee, that fourteen years of age was too young for a child to leave an orphanage, and that it would be an immense advantage to the children and a great strength to the charity if that age was extended to sixteen in the case of boys, so that they might be taught a trade before they were turned out into the world. As to the girls, the amount of manual labour they had to do was, in many instances that had been brought under his notice, too great. Whilst it was desirable that the girls should be trained in housework and every other kind of work which would enable them to become useful members of society, the greatest care should be taken in the course of that training that there should not be too much stress put upon any individual girl.

The Secretary announced that a total contribution of £4,274 had been received as the result of that dinner, and he hoped that the collecting cards the ladies had kindly accepted that evening would bring in a sum sufficient to pay off the whole of the debt to the bankers.

